# Understanding the Reasons for Deferring ART Among Patients Diagnosed Under the Same-Day-ART Policy in Johannesburg, South Africa

**DOI:** 10.1007/s10461-021-03171-6

**Published:** 2021-02-03

**Authors:** Dorina Onoya, Tembeka Sineke, Idah Mokhele, Jacob Bor, Matthew P. Fox, Jacqui Miot

**Affiliations:** 1 Health Economics and Epidemiology Research Office, Department of Internal Medicine, School of Clinical Medicine, Faculty of Health Sciences, University of the Witwatersrand, 39 Empire Road, Empire Park, Parktown, Johannesburg, 2193 South Africa; 2 Department of Epidemiology, Boston University School of Public Health, Boston, MA USA; 3 Departments of Global Health, Boston University School of Public Health, Boston, MA USA

**Keywords:** Ame-day ART initiation, South Africa, ART deferral

## Abstract

We aimed to examine the correlates of antiretroviral therapy (ART) deferral to inform ART demand creation and retention interventions for patients diagnosed with HIV during the Universal Test and Treat (UTT) policy in South Africa. We conducted a cohort study enrolling newly diagnosed HIV-positive adults (≥ 18 years), at four primary healthcare clinics in Johannesburg between October 2017 and August 2018. Patients were interviewed immediately after HIV diagnosis, and ART initiation was determined through medical record review up to six-months post-test. ART deferral was defined as not starting ART six months after HIV diagnosis. Participants who were not on ART six-months post-test were traced and interviewed telephonically to determine reasons for ART deferral. Modified Poisson regression was used to evaluate correlates of six-months ART deferral. We adjusted for baseline demographic and clinical factors. We present crude and adjusted risk ratios (aRR) associated with ART deferral. Overall, 99/652 (15.2%) had deferred ART by six months, 20.5% men and 12.2% women. Baseline predictors of ART deferral were older age at diagnosis (adjusted risk ratio (aRR) 1.5 for 30–39.9 vs 18–29.9 years, 95% confidence intervals (CI): 1.0–2.2), disclosure of intentions to test for HIV (aRR 2.2 non-disclosure vs disclosure to a partner/spouse, 95% CI: 1.4–3.6) and HIV testing history (aRR 1.7 for  > 12 months vs < 12 months/no prior test, 95% CI: 1.0–2.8). Additionally, having a primary house in another country (aRR 2.1 vs current house, 95% CI: 1.4–3.1) and testing alone (RR 4.6 vs partner/spouse support, 95% CI: 1.2–18.3) predicted ART deferral among men. Among the 43/99 six-months interviews, women (71.4%) were more likely to self-report ART initiation than men (RR 0.4, 95% CI: 0.2–0.8) and participants who relocated within SA (RR 2.1 vs not relocated, 95% CI: 1.2–3.5) were more likely to still not be on ART. Under the treat-all ART policy, nearly 15.2% of study participants deferred ART initiation up to six months after the HIV diagnosis. Our analysis highlighted the need to pay particular attention to patients who show little social preparation for HIV testing and mobile populations.

## Introduction

South Africa is home to nearly eight million persons living with HIV, with about 4.6 million of these individuals receiving antiretroviral therapy (ART) [1–3]. While this represents an impressive scale-up effort, ART coverage is still below the targeted 90% by 2020, with about 60% of HIV accessing ART in 2019 [3–5].

Early ART uptake is essential to the success of South Africa’s Universal-Test-and-Treat (UTT) policy [6–8]. The more recent adoption of the WHO strategy to initiate patients on ART immediately after HIV diagnosis regardless of CD4 cell count is expected to reduce late-stage ART initiations, thus reducing HIV-related morbidity and mortality [7, 9–13]. Same-day initiation (SDI) of ART for all HIV infected individuals can potentially reduce HIV incidence by decreasing in the infectious period and overall community viral load [7, 13]. Furthermore, the ART program in South Africa has historically suffered from significant patient losses throughout the care cascade with nearly two-thirds of patients losses occurring between HIV diagnosis and ART initiation [14–16]. Patient-level determinants of these losses included the unclear benefits of pre-ART care, male gender, difficulties with HIV disclosure and stigma, long clinic waiting-time and the high cost of attending clinic visits [17, 18]. Men, in particular, are historically less likely to seek health care or test for HIV, and often enter the care cascade at late stages of the disease, with low retention in care [17–19].

It is logical to expect that removing the CD4-based ART eligibility criteria will reduce the substantial burden of the pre-ART portion of losses from HIV care. However, it is also plausible that currently asymptomatic HIV positive patients (i.e. those with higher CD4 counts) may not appreciate the immediate benefits of starting lifelong HIV treatment [20]. A study conducted between 2008 and 2009, when the ART eligibility in South Africa was CD4 < 200, found that 20% of eligible patients refused to initiate ART, with 92% of these refusing ART up to two months after diagnosis [21, 22]. Still, there is little evidence of high demand for immediate ART among higher CD4 patients, and few studies have determined the magnitude of patients ART deferral under the SDI strategy. The ANRS 12249 TasP trial conducted in rural South Arica, using a home-based HIV testing approach, reported a 33% ART uptake among ART-naïve patients in the "treat-all" randomization arm [23]. These data suggest that overt or passive treatment refusal may impact on early ART uptake and promote early attrition from care under the SDI policy in South Africa. It is, therefore, essential to determine the extent of this phenomenon under the current ART initiation strategy and understand correlates of treatment readiness to inform better ART demand creation and retention interventions for newly diagnosed HIV positive patients.

We aimed to estimate the rate of ART deferral up to six months after HIV diagnosis among newly diagnosed HIV positive patients under the SDI protocol in Johannesburg. We also set out to identify predictors of ART deferral among newly diagnosed HIV positive patients under a UTT protocol in the Gauteng province, South Africa.

## Methods

### Study Setting and Design

We conducted a prospective cohort study enrolling newly diagnosed HIV positive adult (≥ 18 years) participants from October 2017 to August 2018 at four primary healthcare clinics (PHCs) in Johannesburg, South Africa. Participant enrolment co-occurred across sites until the total sample size was attained.

All participants were enrolled in the study by trained interviewers via referral from PHC-based lay HIV counsellors, immediately after HIV diagnosis. Participants were interviewed on the day of enrolment into the study. Participants self-reported being newly diagnosed during the screening process. Enrolled patients with a prior history of ART were excluded from the analytic dataset. We also excluded patients who were psychologically unable or too sick to participate, unwilling to provide consent or planned to get treatment elsewhere. Additionally, women who were pregnant at HIV diagnosis were excluded from the study because in-pregnancy treatment initiation and care processes differ from that of non-pregnant women.

### Data Collection

Eligible patients who provided written consent completed a structured baseline questionnaire on the day of HIV diagnosis. The questionnaire assessed baseline intention to initiate ART, factors that could affect HIV treatment readiness including ART acceptability, socioeconomic status, HIV testing history, food security, experiences with clinic services, alcohol use, HIV knowledge and risk perceptions. The information sheet and questionnaire were available in English, Sesotho and isiZulu, which are widely spoken and understood in Johannesburg.

Participants were followed up via medical record review from the date of HIV diagnosis for six months or until the date of transfer/death in the first six months of care. Clinical information was collected from participants' paper-based and electronic routine medical records, including laboratory test results. Results of baseline laboratory tests (test from first blood samples) were collected one calendar month after enrolment because safety laboratory test results are not available on the day of testing. A telephonic follow-up structured questionnaire was administered to participants who had not initiated ART at six months to establish the reasons for ART deferral/refusal. The questionnaire included an open-ended question about reasons for ART deferral, for those who self-reported not being on ART during the interview. Interviewers made up to three call attempts, on three different occasions, before classifying a person as “Not found”.

### Analytic Variables

The primary outcome was ART deferral (no ART) up to six months after HIV diagnosis. ART deferral percentage was calculated as the number of newly diagnosed HIV infected individuals with no confirmed ART initiation data divided by the total number of newly diagnosed HIV infected individuals who agreed to participate in the study.

We dichotomized participants’ motivation for HIV testing into asymptomatic and symptomatic. Patients who reported testing because of perceived HIV exposure were categorized as asymptomatic. Patients reporting ill-health as the main reason for presenting for HIV testing were regarded as symptomatic. Perceived social support (PSS) was measured using an eight-item scale in which participants indicated their overall satisfaction with available support given in each area (Cronbach’s alpha = 0.61) [24]. Mean scores were categorized as low (< 2), medium (2 to < 3), or high PSS (≥ 3).

We developed a household amenities index assessing participants’ access to 13 desirable household characteristics (flush toilet facilities, electricity, gas energy, brick housing structure, low-medium household density, and food availability), and ownership of durable household assets (television, radio, refrigerator, cellular telephone, landline telephone, microwave oven, and personal computer) (Cronbach’s alpha = 0.79) [25]. Each of the thirteen questions was scored separately on a four-point scale. The total score for the household amenities index ranged from 0 to 1, categorized as low (≤ 4.3), medium (4.4–8.6) or high (≥ 8.7).

Depression was measured using the CES-D 10 scale, a 10-question four-point scale (scores range 0 to 3) that measures general depressive symptoms experienced up to seven days before the interview [26]. The total score ranged from zero to 30, with higher scores reflecting higher depression (Cronbach’s alpha = 0.81). Depression was dichotomized into major depression (total score of  ≥ 12) and no major depression (total score  < 12) [27, 28].

Concerns about ART were measured using a 12-item questionnaire with a 4-point scale ranging from one (Strongly disagree) to four (Strongly agree) (Cronbach’s alpha = 0.83). Participants were asked to respond to statements indicating concerns about ART including “I worry about the long term effects of Antiretroviral medication”, “I worry that Antiretroviral medication will give me unpleasant side effects” and “I feel healthy, I do not see a need to take Antiretroviral medication”. Mean scores were categorized as low (< 2), medium (2–3) and high ART concerns (> 3).

Other socio-demographic factors assessed include age, sex, highest education completed, English literacy, marital status, employment status, whether the patient is the household breadwinner, the number of child dependants and the primary source of income. Factors related to health care access, including healthcare-seeking history, HIV testing history, were also assessed. We also assessed risky sexual behaviours including condom usage at last sex, the number of sexual partners in the previous 12 months and disclosure of intentions to test for HIV, and whether anyone accompanied patients to the testing clinic.

The baseline characteristics of study participants are described using percentages, frequencies, means with standard deviation, and medians with interquartile ranges (IQR) as appropriate. Factors identified with a univariate p-value  < 0.1 and apriori factors of ART deferral such as sex and age were included in the adjusted model. The contribution of baseline CD4 cell count in explaining ART deferral was also assessed using Modified Poisson regression models, reporting Risk Ratios(RR) and 95% confidence intervals (95% CI). All statistical analyses were conducted in STATA 14™ (College Station, TX, USA). The study protocol was reviewed and approved by the Human Research Ethics Committee of the University of Witwatersrand (M1704122).

## Results

### Demographic Characteristics

**Fig. 1:**
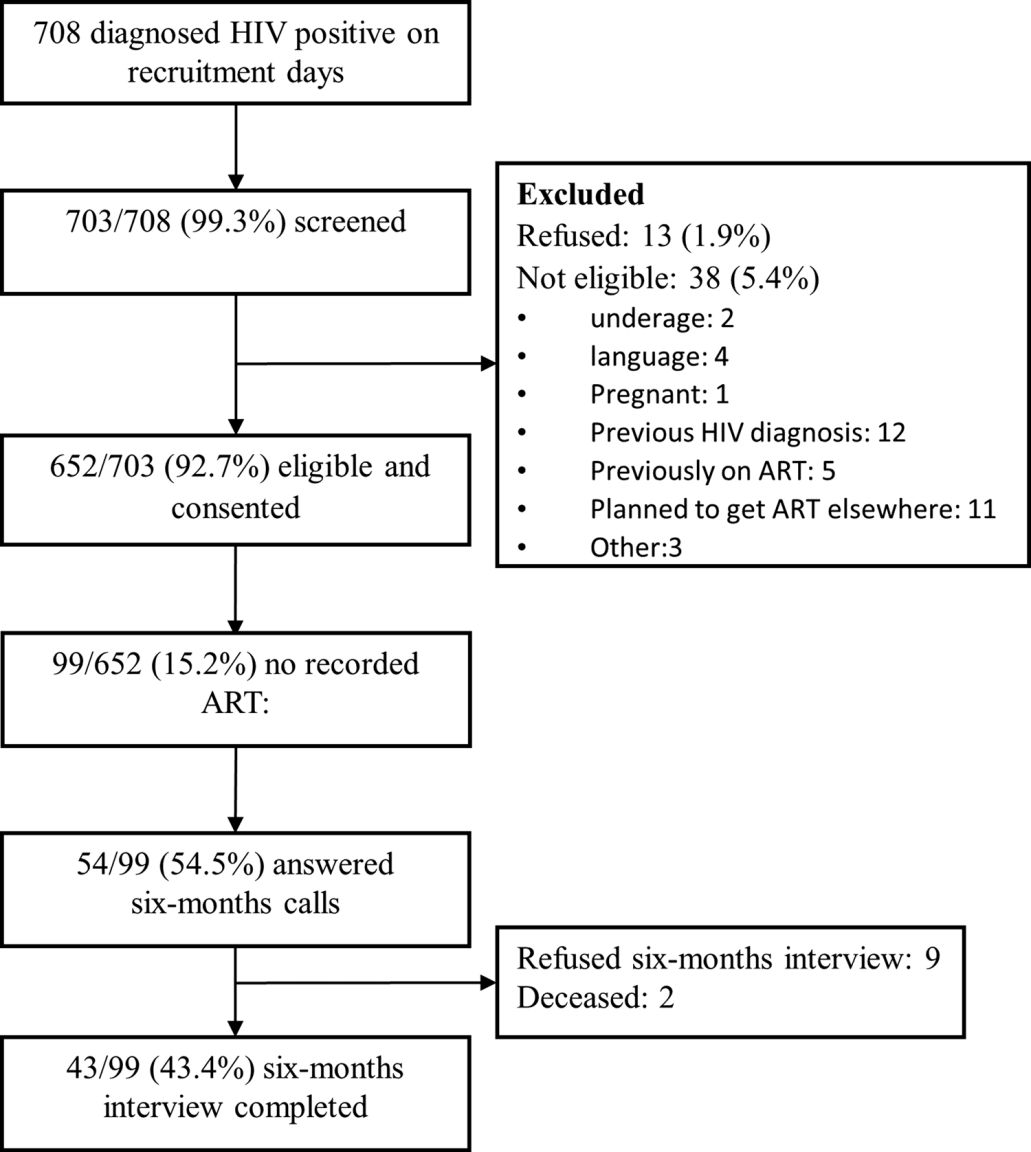
Recruitment, participant eligibility and enrollment of adults (≥ 18 years) at four clinics in Johannesburg

Of the 708 patients who tested positive during the study period,703 (99.3%) newly diagnosed patients were successfully referred and screened, 652 (92.7%) were eligible and agreed to participate in the study (Fig. 1). Overall, the median age at HIV diagnosis was 33 years (IQR:28.0–39.0), and 64.1% of participants were female. The majority (86.0%) had at least a secondary school level of education, 87.5% among women and 83.4% among men. A slightly higher proportion of women (58.9%) reported high levels of English literacy compared to men (47.6%). However, more men (84.0%) reported employment/business as their primary source of income compared to women (51.1%), who were mostly dependent on a partner or family members for financial support.

Overall, 14.1% of participants were married, and 19.5% were not in an active sexual partnership. About a third (31.3%) had at least two sexual partners in the prior year, higher among men (42.1%) than women (25.1%). While 74.4% of the study participants lived with a partner/spouse or family/friends, more men (36.9%) lived alone compared to 18.7% of women.

### Baseline Social Support and Health-Seeking Behaviour (Table 1)

Overall, 53.0% had visited a clinic, and 31.6% had also tested for HIV in the prior 12-months. General clinic attendance in the prior year was lower among men (38.2%) than women (61.4%). More women (36.3%) had tested for HIV in the prior year than men (23.2%). Participants’ assessment of their current social support was medium to high (95.3%), but only 62.4% had disclosed their intention to test for HIV and 26.0% were accompanied to the testing site. Overall disclosure of intentions to test for HIV was similar across gender (65.2% among women compared 57.3% of men). Notably, a lower proportion of men (17.7%) had disclosed to family members/friends compared to women (33.1%). However, participants’ baseline intention to start ART was nearly universal at 99.4%, with only 0.6% planning to never start ART, hence the term ART deferral and not refusal. Similarly, 96.3% of participants intended to disclose their HIV positive status (Table 1).

**Table 1:** Social and demographic characteristics of newly diagnosed patients with HIV (N = 652)

	Female	Male	Total
	No	No	No
Median age at HIV diagnosis, years (IQR)	32 (27.0–38.0)	36 (31.0–42.0)	33 (28.0–39.0)
Age at HIV diagnosis, years			
18–29.99	160 (38.3)	45 (19.2)	205 (31.4)
30–39.99	182 (43.5)	111 (47.4)	293 (44.9)
40+	76 (18.2)	78 (33.3)	154 (23.6)
Education level			
Primary school or less	52 (12.4)	39 (16.7)	91 (14.0)
Some secondary school	243 (58.1)	141 (60.3)	384 (58.9)
≥Grade 12	123 (29.4)	54 (23.1)	177 (27.1)
English literacy			
I can read very well	245 (58.9)	110 (47)	355 (54.6)
I can read somewhat	132 (31.7)	97 (41.5)	229 (35.2)
I cannot read	39 (9.4)	27 (11.5)	66 (10.2)
Primary source of income/finances			
Paid job, salary or business	212 (51.1)	194 (84.0)	406 (62.8)
Spouse/ partner	102 (24.6)	10 (4.3)	112 (17.3)
Parents/relatives/friends/other	101 (24.3)	27 (11.7)	128 (19.8)
Breadwinner of household			
Yes	152 (37.1)	179 (76.8)	331 (51.5)
No	261 (63.7)	54 (23.2)	315 (49.0)
Marital status			
Married	39 (9.4)	53 (22.6)	92 (14.1)
In a relationship (living together)	152 (36.5)	78 (33.3)	230 (35.3)
In a relationship (not living together)	139 (33.3)	63 (26.9)	202 (31.0)
Not in a relationship	87 (20.9)	40 (17.1)	127 (19.5)
Number of sexual partner in the past 12 months			
None	47 (11.5)	17 (7.3)	64 (10.0)
1 partner	260 (63.4)	118 (50.6)	378 (58.8)
≥ 2 partners	103 (25.1)	98 (42.1)	201 (31.3)
Condom use at last sex			
Yes	128 (31.1)	83 (35.6)	211 (32.7)
No	284 (68.9)	150 (64.4)	434 (67.3)
Lives with			
Partner/spouse	181 (49.9)	105 (47.3)	286 (48.9)
Family/friends	114 (31.4)	35 (15.8)	149 (25.5)
Alone	68 (18.7)	82 (36.9)	150 (25.6)
Number of child dependents			
None	222 (54.0)	159 (68.5)	381 (59.3)
1–2 children	145 (35.3)	52 (22.4)	197 (30.6)
≥ 3 children	44 (10.7)	21 (9.1)	65 (10.1)
Primary house			
Current house	144 (35.0)	91 (39.6)	235 (36.7)
Another province/rural	145 (35.3)	80 (34.8)	225 (35.1)
Another country	122 (29.7)	59 (25.7)	181 (28.2)
Housing type			
House/Flat	93 (22.4)	42 (18.1)	135 (20.9)
Cottage/room in backyard	204 (49.2)	102 (44.0)	306 (47.3)
Informal dwelling/shack	118 (28.4)	88 (37.9)	206 (31.8)
Household density			
Low density	112 (27.5)	88 (38.4)	200 (31.4)
Med density	181 (44.4)	99 (43.2)	280 (44.0)
High density	115 (28.2)	42 (18.3)	157 (24.6)
Duration at current house			
Less than 1 year	89 (21.4)	34 (14.6)	123 (19.0)
1–5 years	145 (34.9)	67 (28.8)	212 (32.7)
More than 5 years	182 (43.8)	132 (56.7)	314 (48.4)
Recent clinic attendance (any reason)			
Never	40 (9.7)	67 (28.8)	107 (16.6)
≤ 12 months		89 (38.2)	342 (53.0)
> 12 months	119 (28.9)	77 (33.0)	196 (30.4)
HIV testing history			
≤ 12 months ago	149 (36.3)	54 (23.2)	203 (31.6)
> 12 months ago	202 (49.1)	85 (36.5)	287 (44.6)
Never tested for HIV before current test	60 (14.6)	93 (39.9)	153 (23.8)
Reason for seeking an HIV testing			
Just to know	86 (20.9)	26 (11.2)	112 (17.4)
Current or previous HIV risk	48 (11.7)	48 (20.7)	96 (14.9)
Experiencing symptoms	277 (67.4)	158 (68.1)	435 (67.6)
Perceived social support (PSS)			
Med to high PSS	391 (95.1)	221 (95.7)	612 (95.3)
Low PSS	20 (4.9)	10 (4.3)	30 (4.7)
Disclosed intention to test for HIV			
Partner/spouse	132 (32.1)	92 (39.7)	224 (34.8)
Family/friends/other	136 (33.1)	41 (17.7)	177 (27.5)
No one	143 (34.8)	99 (42.7)	242 (37.6)
Support at clinic for latest HIV test			
Partner/spouse	37 (9.0)	39 (16.8)	76 (11.8)
Family	54 (13.1)	9 (3.9)	63 (9.8)
Other	22 (5.4)	6 (2.6)	28 (4.4)
No one	298 (72.5)	178 (76.7)	476 (74.0)
Intention to disclose HIV test result			
Yes	395 (96.3)	223 (96.5)	618 (96.3)
No	15 (3.7)	8 (3.5)	24 (3.7)
Intention to start ART			
Yes	406 (99.3)	225 (99.6)	631 (99.4)
No	3 (0.7)	1 (0.4)	4 (0.6)
Depression			
No depression	372 (91.0)	205 (91.1)	577 (91.0)
Major depression	37 (9.0)	20 (8.9)	57 (9.0)
Concerns regarding ART			
Low	197 (47.9)	110 (47.4)	307 (47.7)
Medium	214 (52.1)	121 (52.2)	335 (52.1)
High	0	1 (0.4)	1 (0.2)
Baseline CD4 count			
< 350	120 (28.7)	71 (30.3)	191 (29.3)
350–500	38 (9.1)	19 (8.1)	57 (8.7)
> 500	62 (14.8)	10 (4.3)	72 (11.0)
Missing	198 (47.4)	134 (57.3)	332 (50.9)

A large proportion of participants were missing baseline CD4 results (50.9%), more so among men (57.3%) than women (47.4%). Among those who tested for CD4, only 22.5% were diagnosed with HIV at CD4 count  > 500 cells/µl, more so among women (28.2%) than men (10.0%). Overall, 52.1% of participants expressed moderate concerns about ART, with 47.7% expressing low concerns about ART.

### ART Deferral at the Testing Site, Six-Months After HIV Diagnosis (Table 2)

Overall, 99 (15.2%) participants had deferred ART by six months, at the diagnosing clinic. We present crude and adjusted risk ratios (aRR) of baseline factors associated with ART deferral in Table 2. Male gender was associated with higher ART deferral in the crude model (RR 1.7, 95% CI: 1.2–2.4) but not in the adjusted model, including baseline CD4 count. Half of the sample were missing baseline CD4 count, and participants who were missing CD4 count were three times more likely to defer ART than those who had CD4 data (aRR 3.0, 95% CI: 2.0–4.4). There was no difference between CD4 categories in the risk of ART deferral. Missing a baseline CD4 count was not random as, men were more likely to lack baseline CD4 data compared to women (aRR 1.2, 95% CI: 1.0–1.4). Additionally, having tested for HIV more than 12 months prior was associated with missing baseline CD4 data (aRR 1.3 vs  < 12 months, 95% CI: 1.1–1.6).

**Table 2:** Baseline predictors of deferring ART among newly diagnosed patients

	Not initiated on ART (n = 99)	RR	RR
	No. (%)	95% CI	95% CI
Sex			
Female	51 (12.2)	1	1
Male	48 (20.5)	1.7 (1.2–2.4)	1.1 (0.8–1.6)
Age at HIV diagnosis. years			
18–29.9	21 (10.2)	1	1
30–39.9	57 (19.5)	1.9 (1.2–3.0)	1.5 (1.0–2.2)
40+	21 (13.6)	1.3 (0.8–2.3)	1.2 (0.8–2.0)
Marital status			
Married	19 (20.7)	1	
In a relationship (living together)	28 (12.2)	0.6 (0.3–1.0)	
In a relationship (not living together)	31 (15.3)	0.7 (0.4–1.2)	
Not in a relationship	21 (16.5)	0.8 (0.5–1.4)	
Education level			
Primary school or less	16 (17.6)	1	
Some secondary school	64 (16.7)	0.9 (0.6–1.6)	
≥Grade 12	19 (10.7)	0.6 (0.3–1.1)	
English literacy			
I can read very well	44 (12.4)	1	1
I can read somewhat	43 (18.8)	1.5 (1–2.0)	1.2 (0.9–1.7)
I cannot read	12 (18.2)	1.5 (0.8–2.6)	0.9 (0.5–1.6)
Primary house			
Current house	22 (9.4)	1	1
Another province/rural	34 (15.1)	1.6 (0.9–2.7)	1.6 (1.1–2.3)
Another country	41 (22.7)	2.4 (1.5–3.9)	2.1 (1.4–3.1)
Primary source of income/finances			
Paid job. salary or business	63 (15.5)	1	
Spouse/partner	15 (13.4)	0.9 (0.5–1.5)	
Parents/ relatives/friends/other	18 (14.1)	0.9 (0.6–1.5)	
Breadwinner of household			
Yes	50 (15.1)	1	
No	47 (14.9)	1.0 (0.7–1.4)	
Lives with			
Partner/spouse	37 (12.9)	1	
Family/friends	24 (16.1)	1.2 (0.8–2.0)	
Alone	25 (16.7)	1.3 (0.8–2.1)	
Number of child dependents			
None	55 (14.4)	1	
1–2 children	17 (14.0)	1.0 (0.6–1.6)	
≥3 children	27 (18.0)	1.2 (0.8–1.9)	
Last visit to any health provider			
Never	21 (19.6)	1	
Within a year	51 (14.9)	0.8 (0.5–1.2)	
More than a year ago	25 (12.8)	0.6 (0.4–1.1)	
Ever visited current clinic			
Yes	37 (14.3)	1	
No	39 (13.9)	1.0 (0.6–1.5)	
Last HIV test before current test			
≤12 months ago	19 (9.4)	1	1
> 12 months ago	49 (17.1)	1.8 (1.1–3.0)	1.3 (0.9–2.0)
Never tested for HIV before current test	27 (17.6)	1.9 (1.1–3.3)	1.3 (0.8–2.0)
Condom use at last sex			
Yes	35 (16.6)	1	
No	62 (14.3)	0.9 (0.6–1.3)	
Number of sexual partner in the past 12 months			
None	9 (14.1)	1	
1 partner	59 (15.6)	1.1 (0.6–2.1)	
2+ partners	29 (14.4)	1.1 (0.5–2.1)	
Reason for seeking an HIV testing			
Just to know	10 (8.9)	1	1
Current or previous HIV risk	11 (11.5)	1.3 (0.6–2.9)	1.0 (0.5–2.0)
Experiencing symptoms	73 (18.0)	2.0 (1.1–3.8)	1.2 (0.7–2.0)
Depression			
No depression	81 (14.0)	1	
Major depression	12 (21.1)	1.5 (0.9–2.6)	
Perceived social support			
Medium to high	90 (14.7)	1	
Low	4 (13.3)	0.9 (0.4–2.3)	
Intention to disclose			
Yes	91 (14.7)	1	
No	3 (12.5)	0.8 (0.3–2.5)	
Disclosed intention to test			
partner/spouse	21 (9.4)	1	1
Family/friends/other	16 (9.0)	1.0 (0.5–1.8)	1.1 (0.7–1.9)
No one	58 (24)	2.6 (1.6–4.1)	1.9 (1.3–2.8)
Concerns regarding ART			
Low	54 (17.6)	1	
Medium	68 (20.3)	1.2 (0.8–1.6)	
High	1 (100)	–	
Support at clinic for latest HIV test			
Partner/spouse	8 (10.5)	1	
Family/other	14 (15.4)	1.5 (0.6–3.3)	
No one	73 (15.3)	1.5 (0.7–2.9)	
Baseline CD4			
< 350	18 (9.4)	1	
350–500	6 (10.5)	1.1 (0.5–2.7)	1.3 (0.6–3.1)
> 500	4 (5.6)	0.6 (0.2–1.7)	0.7 (0.2–2.0)
Missing	98 (29.5)	3.1 (2.0–5.0)	3.0 (1.8–4.7)

Participants who were 30–39 years old were 50% more likely to defer ART compared to the younger (18–30 years) group (aRR 1.5, 95% CI: 1.0–2.2). However, men were also older than women at testing (RR 1.72 for men being 30–39 years old vs 18–30 years, 95% CI: 1.3–2.3; RR 2.3 for being  ≥ 40 vs 18–30 years, 95% CI: 1.7–3.2).

Participants who tested more than 12 months before study enrolment were more likely to defer ART compared to those who tested more recently (aRR 1.7, 95% CI:1.0–2.8). However, recent risk behaviour such as unprotected sex and multiple sexual partnerships in the prior 12 months did not predict ART uptake. Also, participants who did not disclose their intention to test for HIV (aRR 2.2 for non-disclosure vs disclosure to a partner/spouse, 95% CI: 1.4–3.6) were more likely to defer HIV treatment.

Although 52.1% participants expressed moderate concerns about ART, the association between reported concerns about ART with ART deferral was weak and imprecise (aRR 1.2 for moderate vs low, 95% CI: 0.9–2.3). Participants who reported having a primary house outside South Africa were twice more likely to defer ART compared to local participants (aRR 2.1, 95% CI: 1.0–4.6).

### Baseline Predictors of ART Deferral Among Newly Diagnosed Male Participants (Table 3)

In the analysis restricted to male participants, reporting only crude associations due to the small sample size (n = 234), older age at HIV diagnosis remained an important predictor of ART deferral. Social preparations for testing such as telling a partner about intentions to test (RR 3.3 for not disclosing vs telling a partner, 95% CI: 1.7–6.6) and having support at the testing site (RR 4.6 no support vs being accompanied by a partner, 95% CI: 1.2–18.3) were important predictors of ART deferral among men. Similarly, men who lived with family/friends rather than a partner/spouse (RR 2.1, 95% CI: 1.0–4.0) and those who had never tested before (RR 1.9 vs  < 12 months, 95% CI: 1.1–3.3) or tested more than 12-months prior (RR 1.8 vs < 12 months, 95% CI: 1.1–3.0) were more likely to defer ART.

**Table 3:** Baseline predictors of ART deferral among newly diagnosed male patients

	Not initiated on ART (n = 48)	RR
	No. (%)	95% CI
Age at HIV diagnosis. years		
18–29.99	5 (11.1)	1
30–39.99	29 (26.1)	2.4 (1.0–5.7)
40+	14 (17.9)	1.6 (0.6–4.2)
Marital status		
Married	12 (22.6)	1
In a relationship (living together)	12 (15.4)	0.7 (0.3–1.4)
In a relationship (not living together)	15 (23.8)	1.1 (0.5–2.0)
Not in a relationship	9 (22.5)	0.9 (0.5–2.1)
Education level		
Primary school or less	11 (28.2)	1
Some secondary school	29 (20.6)	0.7 (0.4–1.3)
≥ Grade 12	8 (14.8)	0.5 (0.2–1.2)
English literacy		
I can read very well	18 (16.4)	1
I can read somewhat	22 (22.7)	1.4 (0.8–2.4)
I cannot read	8 (29.6)	1.8 (0.9–3.7)
Primary house		
Current house	11 (12.1)	1
Another province/rural	16 (20.0)	1.7 (0.8–3.4)
Another country	20 (33.9)	2.8 (1.4–5.4)
Primary source of income/finances		
Paid job salary or business	38 (19.6)	1
Spouse/ partner	2 (20.0)	0.9 (0.3–3.7)
Parents/relatives/friends/other	6 (22.2)	1.1 (0.5–2.4)
Breadwinner of household		
Yes	31 (17.3)	1
No	16 (29.6)	1.7 (1.0–2.9)
Lives with		
Partner/spouse	16 (15.2)	1
Family/friends	11 (31.4)	2.1 (1.0–4.0)
Alone	16 (19.5)	1.3 (0.7–2.4)
Number of child dependents		
None	29 (18.2)	1
1–2 children	9 (28.1)	1.5 (0.8–2.9)
≥3 children	10 (23.3)	1.3 (0.7–2.4)
Last visit to any health provider		
Never	16 (23.9)	1
Within a year	17 (19.1)	0.8 (0.4–1.5)
More than a year ago	14 (18.2)	0.8 (0.4–1.4)
Ever visited current clinic		
Yes	13 (17.6)	1
No	18 (19.6)	1.1 (0.6–2.1)
Last HIV test before current test		
≤ 12 months ago	9 (16.7)	1
> 12 months ago	21 (24.7)	1.8 (1.1–3.0)
Never tested for HIV before current test	16 (17.2)	1.9 (1.1–3.3)
Condom use at last sex		
Yes	17 (20.5)	1
No	30 (20.0)	1.0 (0.6–1.7)
Number of sexual partner in the past 12 months		
None	3 (17.6)	1
1 partner	25 (21.2)	1.2 (0.4–3.6)
2+ partners	19 (19.4)	1.1 (0.4–3.3)
Reason for seeking an HIV test		
Just to know	1 (3.8)	1
Current or previous HIV risk	5 (10.4)	2.7 (0.3–22.1)
Experiencing symptoms	41 (26.5)	6.9 (0.9–48.1)
Depression		
No depression	37 (18.0)	1
Major depression	7 (35.0)	1.9 (0.9–3.8)
Perceived social support		
Medium to high	43 (19.5)	1
Low	2 (20.0)	1.0 (0.3–3.7)
Intention to disclose HIV positive diagnosis		
Yes	43 (19.3)	1
No	2 (25.0)	1.3 (0.4–4.4)
Disclosed intention to test for HIV		
Partner/spouse	9 (9.8)	1
Family/friends/other	5 (12.2)	1.2 (0.4–3.5)
No one	32 (32.3)	3.3 (1.7–6.6)
Concerns regarding ART		
Low	21 (19.1)	1
Medium	33 (27.3)	1.4 (0.9–2.3)
High	1 (100)	–
Support at clinic for latest HIV test		
Partner/spouse	2 (5.1)	1
Family	2 (13.3)	2.6 (0.4–16.9)
No one	42 (23.6)	4.6 (1.2–18.3)
Baseline CD4 (up to 30 post-test)		
< 350	7 (9.9)	1
350–500	1 (5.3)	0.5 (0.1–4.1)
> 500	1 (10.0)	1.0 (0.1–7.4)
Missing	48 (35.8)	3.6 (1.7–7.6)

While employment status did not predict ART deferral, being the household breadwinner was associated with a higher risk of not starting ART (RR 1.7, 95% CI: 1.0–2.9). Also, those who lived in temporary homes (RR 2.8 when the primary house is in another country vs the current house, 95% CI: 1.4–5.4) were more likely to defer ART.

### Reason for Deferring ART Initiation Among Newly Diagnosed Participants (Table 4)

Of the 99 (15.2%) participants who did not take-up ART at the testing site, 54 (54.5%) were successfully traced at six-months post-test. Of the 54 traced participants, two were deceased, nine refused to be interviewed, and 43 (43.4%) completed the follow-up telephonic interview. Among those interviewed, 27 (62.8%) reported having started ART, half (51.9%) of these reported having started ART at the diagnosing site. Women (71.4%) were more likely to self-report ART initiation than men (26.9%, RR 0.4 men vs women, 95% CI: 0.2–0.8). Among those interviewed at six months, relocation to other provinces within South Africa was the main reason for not starting ART (RR 2.1 vs no move, 95% CI: 1.2–3.5). Only three (7.7%) of those interviewed at six-months reported high ART concerns. ART concerns did not influence participants’ decision to start ART (RR 0.9 for medium vs low concerns, 95% CI: 0.6–1.4).

**Table 4:** Predictors of self-reported ART deferral at six-months post-test among participants with no documented ART at the diagnosing clinic

	Women	Men	Total	No ART at six months
	N = 24	N = 19	N = 43	
	n (%)	n (%)	n (%)	RR (95 % CI)
Did you move from your house in the last 6 months?				
Yes	14 (58.3)	12 (63.2)	26 (60.5)	1.6 (0.9–2.7)
No	10 (41.7)	7 (36.8)	17 (39.5)	1
New location of those who moved				
Yes, other country	1 (5.0)	1 (5.9)	2 (5.4)	1.1 (0.2–4.7)
Yes, other province in SA	3 (15.0)	0	3 (8.1)	2.1 (1.2–3.5)
Yes, other town/suburb in Gauteng	6 (30.0)	9 (52.9)	15 (40.5)	1.4 (0.8–2.7)
No relocation	10 (50.0)	7 (41.2)	17 (48.6)	1
Self-reported ART start at six months				
Yes	20 (83.3)	7 (36.8)	27 (62.8)	
No	4 (16.7)	12 (63.2)	16 (37.2)	
ART start at six months at diagnosing clinic				
Yes	10 (50.0)	4 (57.1)	14 (51.9)	
No	10 (50.0)	3 (42.9)	13 (48.1)	
ART concerns at six months				
High ART concerns	1 (5.0)	2 (10.5)	3 (7.7)	0.5 (0.1–2.4)
Medium ART concerns	9 (45.0)	12 (63.2)	21 (53.8)	0.9 (0.6–1.4)
Low ART concerns	10 (50.0)	5 (26.3)	15 (38.5)	1
Depression at six months				
No depression	12 (92.3)	12 (92.3)	24 (92.3)	1
Major depression	1 (7.7)	1 (7.7)	2 (7.7)	0.8 (0.2–3.4)
Perceived social support at six months				
Low	4 (26.7)	3 (23.1)	7 (25.0)	1
Medium	11 (73.3)	10 (76.9)	21 (75.0)	0.9 (0.5–1.6)

Open-ended questions provided qualitative reasons for ART deferral from the 16 interviewed participants who had not started ART. As highlighted by the quotes below, important barriers to ART were high mobility, uncertainties about the procedure for initiating or continuing treatment when the process was interrupted by a move or other social circumstances.

“I have not started my medication yet. I had an emergency in my home country, lost my son in Zimbabwe and was there for five months. I left the country before they could initiate me on treatment, but I am now back but currently in Durban.” – Male participant.

“I moved back home to Bulawayo (Zimbabwe) because my mother was sick, after starting ART, but how will I continue with my treatment now that I have moved?”—Female participant.

“I have not started ART because I was sick and hospitalized and then moved back home to recuperate. I have just moved back to my current house recently.” – Female participant.

## Discussion

This is one of the first studies to explore reasons for ART deferral under the new same-day ART initiation policy in South Africa. Overall, 15.2% of participants had deferred ART by at least six months, 20.5% for men and 12.2% for women. The substantial decline in ART deferral among patients who tested for HIV is consistent with the previous findings in South Africa [1–3]. Although facility-based studies show substantial improvement in linkage to ART, national estimates still show much lower ART coverage, indicating a large gap in linkage to ART among patients tested at primary care facilities compared to total tested, including community-based testing [1–3, 29–31]. Further improvements in the linkage to ART and retention in HIV care of individuals who access HIV testing services at community sites will likely have a more significant impact on overall national ART initiation figures and the achievement of the UNAIDS targets [12].

Although SDI policy was implemented at all health facilities in South Africa, implementation varied between study sites, possibly due to variations in facilities and provider challenges [32]. It is also possible that the limited policy implementation guidance, early on, led to slow assimilation of the UTT and SDI policies [33]. There is, therefore, a need for further engagement to address the challenges that patients and health providers are facing to increase ART uptake and retention in care.

A key finding of this study is that disclosure of plans to get an HIV test and even being accompanied at the testing site reduced ART deferral. Disclosure of one’s intention to test for HIV is a vital step in paving the way for the eventual disclosure of the HIV test results and long term ART adherence. These preparatory steps allow individuals to address concerns about confidentiality, partner and community-level HIV stigma before the receipt of an HIV positive diagnosis [34]. These factors were particularly important among male participants and may be essential intervention targets to decrease the proportion of undiagnosed patients and improve linkage and retention of male patients [35]. Similarly, patients’ familiarity with clinic processes and established healthcare-seeking behavior among men may promote early HIV testing and linkage to ART. In addition to the effect of migration and mobility on the risk of disengagement from care, work commitment for breadwinners, HIV disclosure to family members as well as social preparations for HIV care are essential in patient linkage and retention in HIV care [36, 37]. Similar to other studies in South Africa, participants who deferred ART by six months were generally older at HIV diagnosis compared to those who started ART, male and of non-South African origin [37, 38]. The potential for migration and mobility was a particularly important predictor of not starting ART, among both male and female participants [37, 38].

While employment status did not predict ART deferral, males participants who were primary household income earners were at higher risk of not starting ART [37–41]. Migration often varies by region, affecting more men than women. Labour-related mobility is associated with increased risk of HIV acquisition among both men and women. [38]. Language barriers and poor communication about healthcare administrative processes in the host country commonly hinder the retention of migrants in ART programs [39–41]. Our findings, therefore, echo previous calls for an integrated health information system to accommodate patient mobility needs and facilitate access to care [36].

Previous studies reported a 20% ART deferral rate among ART eligible patients diagnosed under the CD4 < 350 policy [21]. Although the proportion of participants diagnosed with CD4 > 350 was marginally higher during the SDI policy than under previous ART guidelines; the majority still test at CD4 counts below 350 cell/µl [42]. The reduced dependence on CD4 test results for ART initiation under the UTT and SDI policies may have inadvertently resulted in a reduced availability of baseline CD4 results, as nearly half of the participants were missing baseline CD4 data in their clinic files [37]. However, as observed before UTT, participants who did not engage with post-HIV diagnosis processes such as providing blood for baseline tests were by far more likely to defer ART [17, 18, 31].

## Limitation

These study results are limited to the geographic location of the participating primary care facilities. The inclusion of patients who test within non-clinical community settings may have provided more clarity on the reason for ART deferral among diagnosed HIV positive patients who do not link to ART at the local clinic. Also, we only included four facilities in the Johannesburg sub-district A, which may not necessarily reflect the variability in ART deferral and its determinants in South Africa. While the open-ended questions on reasons for ART deferral provided some insight, an appropriately powered study targeting patients who have deferred ART is needed to further inform supportive interventions.

Furthermore, traced participants may have been more inclined to engage about their healthcare-seeking patterns, and may have reported initiating ART because it was a desirable study outcome. Further studies are needed to understand barriers to ART uptake among patients who cannot be contacted and those who make no attempt to visit a PHC after HIV testing. A majority of those who could not be traced could have also been silent refusals or changed clinics for ART initiation. However, South African currently lack a unique patient identifier, and HIV management data are not stored in a networked information system, limiting inter-clinic patient searches.

## Conclusion

Under the treat-all ART policy, 15% of the study participants defer ART initiation up to six months after the HIV diagnosis. Our analysis highlighted the need to pay particular attention to patients who show little social preparation for HIV testing and mobile populations. These factors should be considered in interventions aiming to improve earlier linkage ART among patients who attend primary care facilities in South Africa.
